# Combination of Pneumococcal Surface Protein A (PspA) with Whole Cell Pertussis Vaccine Increases Protection Against Pneumococcal Challenge in Mice

**DOI:** 10.1371/journal.pone.0010863

**Published:** 2010-05-27

**Authors:** Maria Leonor S. Oliveira, Eliane N. Miyaji, Daniela M. Ferreira, Adriana T. Moreno, Patricia C. D. Ferreira, Fernanda A. Lima, Fernanda L. Santos, Maria Aparecida Sakauchi, Célia S. Takata, Hisako G. Higashi, Isaías Raw, Flavia S. Kubrusly, Paulo L. Ho

**Affiliations:** 1 Centro de Biotecnologia, Instituto Butantan, São Paulo, São Paulo, Brazil; 2 Divisão Bioindustrial, Instituto Butantan, São Paulo, São Paulo, Brazil; Hannover School of Medicine, Germany

## Abstract

*Streptococcus pneumoniae* is the leading cause of respiratory acute infections around the world. In Latin America, approximately 20,000 children under 5 years of age die of pneumococcal diseases annually. Pneumococcal surface protein A (PspA) is among the best-characterized pneumococcal antigens that confer protection in animal models of pneumococcal infections and, as such, is a good alternative for the currently available conjugated vaccines. Efficient immune responses directed to PspA in animal models have already been described. Nevertheless, few low cost adjuvants for a subunit pneumococcal vaccine have been proposed to date. Here, we have tested the adjuvant properties of the whole cell *Bordetella pertussis* vaccine (wP) that is currently part of the DTP (diphtheria-tetanus-pertussis) vaccine administrated to children in several countries, as an adjuvant to PspA. Nasal immunization of BALB/c mice with a combination of PspA5 and wP or wP_low_ – a new generation vaccine that contains low levels of *B. pertussis* LPS – conferred protection against a respiratory lethal challenge with *S. pneumoniae*. Both PspA5-wP and PspA5-wP_low_ vaccines induced high levels of systemic and mucosal antibodies against PspA5, with similar profile, indicating no essential requirement for *B. pertussis* LPS in the adjuvant properties of wP. Accordingly, nasal immunization of C3H/HeJ mice with PspA5-wP conferred protection against the pneumococcal challenge, thus ruling out a role for TLR4 responses in the adjuvant activity and the protection mechanisms triggered by the vaccines. The high levels of anti-PspA5 antibodies correlated with increased cross-reactivity against PspAs from different clades and also reflected in cross-protection. In addition, passive immunization experiments indicated that antibodies played an important role in protection in this model. Finally, subcutaneous immunization with a combination of PspA5 with DTP_low_ protected mice against challenge with two different pneumococcal strains, opening the possibility for the development of a combined infant vaccine composed of DTP and PspA.

## Introduction

Recent reports on *Streptococcus pneumoniae* burden worldwide indicate that the health problems caused by this pathogen are far from being solved [1], [2]. The number of deaths caused by pneumococcal diseases is still high in young children, reaching about 1 million cases per year. Around 60% of these deaths occur in developing countries [1]. The introduction of the seven-valent pneumococcal conjugate vaccine (PCV7) has substantially prevented the incidence of pneumococcal invasive diseases caused by vaccine serotypes in developed countries such as the United States, France and Canada [3], [4] with extended benefits to non-immunized individuals by the herd-immunity.

However, the introduction of the PCV7 also highlighted a potential drawback of polysaccharide-based vaccines that is the substitution of the prevalent serotypes for others that were not included in the vaccine. An example of such effect is the emergence of the serotype 19A as a prevalent cause of pneumococcal diseases in countries where the PCV7 has been introduced [5], [6], [7]. Inclusion of additional serotypes in available conjugate vaccines is a natural step forward to the development of new vaccines and is the rationale for the new 10- and 13- valent versions of conjugated vaccines that are under process of licensing. Certainly, serotype coverage displayed by these vaccines will vary depending on the region of the world [8], [9], [10]; but major concerns are related to the possibility of additional serotype substitution, as has occurred within a few years of PCV7 use, and the high costs of conjugated vaccines, especially when they are considered for use in developing countries.

In the past years, several protein antigens have been proposed as vaccine candidates aiming at the development of effective broad-coverage formulations at low costs [11]. Among them, the Pneumococcal surface protein A (PspA) is possibly the most well studied. As a virulence factor, PspA has been implicated in evasion from the immune system by inhibition of complement deposition on bacterial surface [12], [13] and binding to the mucosal bactericidal protein apolactoferrin [14]. Several vaccine formulations based on PspA have proven to be effective in animal models of pneumococcal infections. Vaccine approaches include DNA vaccines [15], [16], [17], antigen delivery by salmonella [18], [19] and lactic-acid bacteria vectors [20], [21], [22] or combination of recombinant proteins with Toll-like receptors agonists or cytokines [23], [24]. Taken together, the results of these publications indicate a strong correlation between increased protection in animal models and the induction of Th1 responses, characterized by high levels of anti-PspA IgG2a and IFN-γ production.

IL-17 secretion by CD4^+^ T lymphocytes was also shown to be an important branch of innate [25] and acquired immune responses to pneumococcal infection induced by a cellular vaccine [26] or vaccines based on pneumococcal cell wall polysaccharide and recombinant proteins [27], [28], [29], [30]. Using a PspA nasal vaccine, we have also shown a correlation between increased protection and secretion of IL-17 by lung and spleen cells, after a lethal respiratory challenge in mice [22].

Research on adjuvants is a crucial matter of vaccine development. Aluminum salts (Alum) have proven to be efficacious for recombinant-protein based formulations in which Th2 responses are very effective. Still, several groups are focusing their studies in describing new adjuvants for applications where cell mediated immunity is desired [31]. Such responses are usually induced by cellular vaccines, as the case of whole cell pertussis vaccine (wP) [32], [33], [34], [35]. In addition to the high efficacy of wP against whooping cough, numerous works have described its efficacy as adjuvant when administered in combination with different antigens [36], [37]. Cellular pertussis vaccines have sometimes been associated with serious side effects in children. Although causal relationship between wP vaccination and side effects has not been well established [38], some countries adopted acellular formulations that induce mild side effects but sometimes display slightly reduced efficacy when compared with wP and have higher production costs [38]. An increase in pertussis cases all over the world has been observed in the recent years [39]. Proposed alternatives for the available vaccines include administration of wP through mucosal routes [40] as well as the use of attenuated *B. pertussis* mutants [41].

In Brazil, more than 260 million doses of DTPw (Diphteria-Tetanus-Whole Cell Pertussis) produced by Instituto Butantan (São Paulo, Brazil) have been administered to children all over the country since 1996, with no reports of serious side effects. In fact, cost-effectiveness and safety results led the Pan American Health Organization (PAHO) to conclude that there is no reason for Latin American countries to change from wP to acellular formulations [42].

In this work, we have hypothesized that a combination of PspA with wP could trigger protective immune responses against pneumococcal infections. A concern related with pneumococcal vaccines composed of PspA is the variability of the protein in pneumococcal isolates, which is the basis for its classification into 6 clades and 3 families [43]. Recently two studies from our group have demonstrated that some fragments can induce antibodies with cross-reactivity among PspAs from different clades and isolates, reflecting in broad coverage vaccine formulations [44], [45]. The experiments presented here were conducted with one of such molecules, PspA5, and two different wP vaccines. The first one, wP, is currently part of the Brazilian DTPw vaccine; the second, is a new generation wP vaccine that contains low levels of LPS (wP_low_) and has recently undergone a phase 1 clinical trial [46]. Local and systemic immune responses were evaluated in mice immunized through the nasal route with both PspA5-wP and PspA5-wP_low_ formulations and challenged with an invasive pneumococcal strain. The role of LPS in the adjuvant activity of wP and the role of anti-PspA antibodies in protection were analyzed. Combined vaccines were also tested for protection against strains expressing heterologous PspAs. Finally, the potential of a combined PspA5-DTP_low_ vaccine in mice was also assessed.

## Results

### Nasal immunization of mice with PspA5 in combination with wP protects mice from pneumococcal infection

BALB/c mice were immunized through the nasal route with PspA5 alone or in combination with wP (PspA5-wP) and 21 days later, were submitted to a respiratory challenge with a lethal dose of the ATCC6303 pneumococcal strain. In this model, non-immunized mice usually die 72 h after challenge [47]. As shown in Table 1, significant protection was observed in the group immunized with the PspA5-wP formulation, whereas 50% protection was observed in mice immunized with PspA5 alone. No protection was observed for non-immunized mice or mice inoculated with wP.

**Table 1:** Survival of BALB/c mice after intranasal challenge with *S. pneumoniae* ATCC6303 - adjuvant properties of wP.

	alive/total	% of survival	*P* *
Non-immunized	0/6	--	
wP	1/6	16.6	1
PspA5	3/6	50	0.09
PspA5-wP	6/6	100	0.001

* Fisher exact test. Results are representative of three independent experiments.

The PspA5-wP vaccine, which induced 100% survival in mice, was chosen for further studies using mice immunized with wP or non-immunized mice as controls. Groups of 4 BALB/c mice were immunized with each of these formulations through the nasal route and then submitted to the respiratory challenge with the ATCC6303 pneumococcal strain.

**Figure 1:**
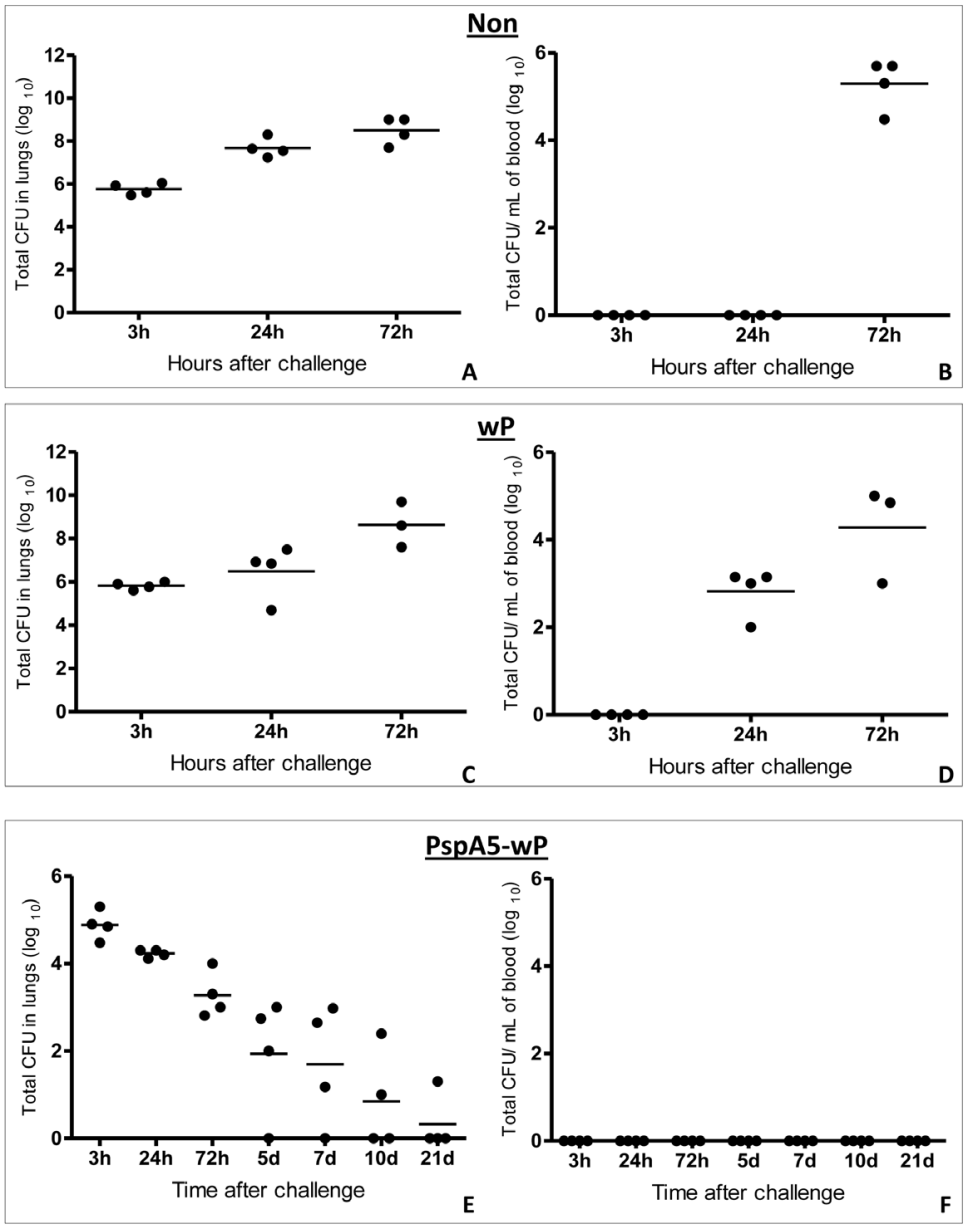
Pneumococcal loads in lungs and sera. Lungs (A, C and E) and sera (B, D and F) from non-immunized BALB/c mice (Non) or mice immunized with wP or PspA5-wP were collected at different time points after intranasal challenge with ATCC6303 pneumococcal strain (PspA5). CFU were determined in four mice per group, after plating the samples in blood-agar. Circles represent individual mice and lines represent the mean for each group. (d = days). In conditions where no bacteria were detected, 1 CFU was considered.

Pneumococcal loads in lung homogenates and blood and were determined at different time points after challenge. As observed in Figure 1, 3 h after challenge, pneumococcal counts were around 10^5^ to 10^6^ bacteria per lung homogenate. These numbers progressively increased in non-immunized mice (A) or mice immunized with wP (C), reaching a maximum around 10^7^ to 10^9^ bacteria per lung homogenate at 72 h. In contrast, after the first time point at 3 hours, the numbers of pneumococci in the lungs of mice immunized with PspA5-wP decreased continuously until the bacteria were almost completely cleared at 21 days after the challenge (E). High number of pneumococci could be recovered from the blood of non-immunized mice or mice immunized with wP, 72 h after challenge (Figure 1B and D, respectively). These mice died few hours later. Immunization with PspA5-wP prevented pneumococcal invasion to bloodstream (Figure 1F).

### Protection against pneumococcal respiratory infection can also be induced by combination of PspA5 with wP_low_

Since a new generation of cellular pertussis vaccine, with low LPS content (wP_low_), was shown to induce similar immune responses against pertussis antigens when compared with conventional wP in a phase 1 clinical trial [46], we decided to test this formulation in combination with PspA5 against the respiratory challenge with *S. pneumoniae* ATCC6303 in mice. Mice were immunized through the nasal route, following the same protocol used for PspA5-wP. As observed in Table 2, significant protection against pneumococcal infection was observed only for the group vaccinated with PspA5-wP_low_ (83.3% survival, *P* = 0.007, when compared with non-immunized mice). Once again, nasal immunization with PspA5 induced partial protection, with 50% of mice survival. Time course experiments performed with the PspA5-wP_low_ vaccine produced results similar to those observed for PspA5-wP. While pneumococcal loads in the lungs of non-immunized mice or mice immunized with wP_low_ tended to increase with time post-challenge, the levels observed in mice immunized with PspA5- wP_low_, progressively decreased. In addition, the PspA5-wP_low_ vaccine also prevented bacteria invasion to the bloodstream (data not shown).

**Table 2:** Survival of BALB/c mice after intranasal challenge with *S. pneumoniae* ATCC6303 – adjuvant properties of wP_low_.

	alive/total	% of survival	*P* *
Non-immunized	0/6	---	---
wP_low_	0/5	---	1
PspA5	3/6	50	0.09
PspA5-wP_low_	5/6	83.3	0.007

* Fisher exact test. Results are representative of two independent experiments.

### PspA5-wP and PspA5wP_low_ vaccines induce high levels of systemic and mucosal anti-PspA5 antibodies

**Figure 2:**
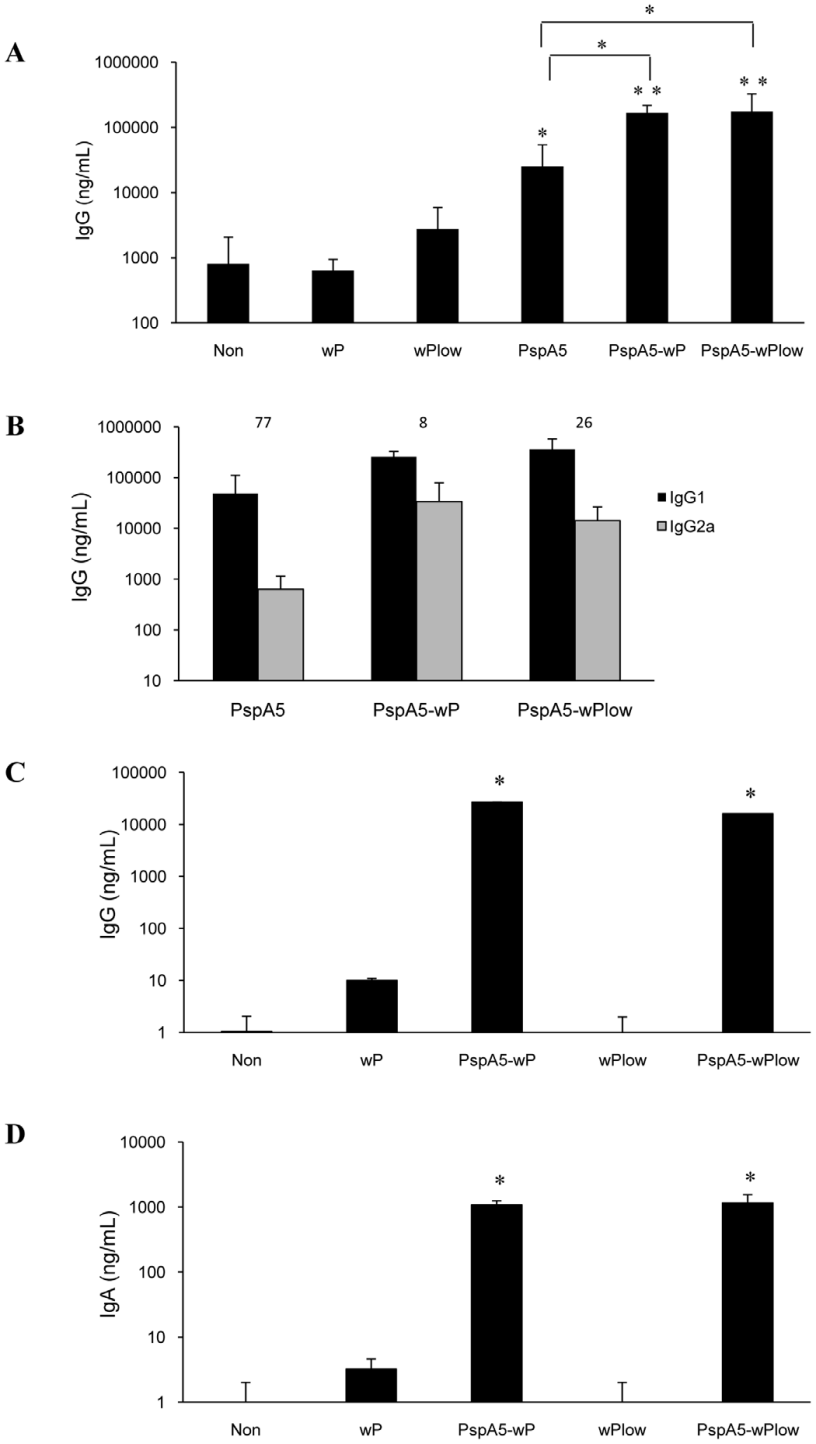
Evaluation of anti-PspA5 antibodies in mice immunized with the different vaccine formulations. Three weeks after the last immunization anti-PspA5 IgG (A), IgG1 and IgG2a (B) in the sera and IgG (C) and IgA (D) in BALF were detected through ELISA. Concentration of antibodies (mean of 6 animals for A and B and 4 animals for C and D) are shown. IgG1∶IgG2a ratios are indicated above the bars (B). Results are representative of two experiments (A and B). Asterisks represent significant differences from control groups or from group immunized with PspA5, when indicated (* *P*<0.05; ** *P*<0.005, Mann-Whitney *U* test).

Nasal immunization with PspA5 induced significant higher levels of anti-PspA5 IgG in the sera of BALB/c mice when compared with control groups. Still, the levels of anti-PspA5 IgG induced by both PspA5-wP and PspA5-wP_low_ formulations were even higher and also significantly different from the observed in mice immunized with PspA5 alone (Figure 2A). Combination of PspA5 with wP and wP_low_ favored the induction of relative higher levels of IgG2a, resulting in lower IgG1∶IgG2a ratios when compared with PspA5 alone (Figure 2B). In addition, both PspA5-wP and PspA5-wP_low_ formulations induced high levels of anti PspA5 IgG and IgA in BALF, as observed in Figures 2C and 2D, respectively.

### Protection induced by the PspA5-wP vaccine is not dependent on TLR4 activation

**Figure 3:**
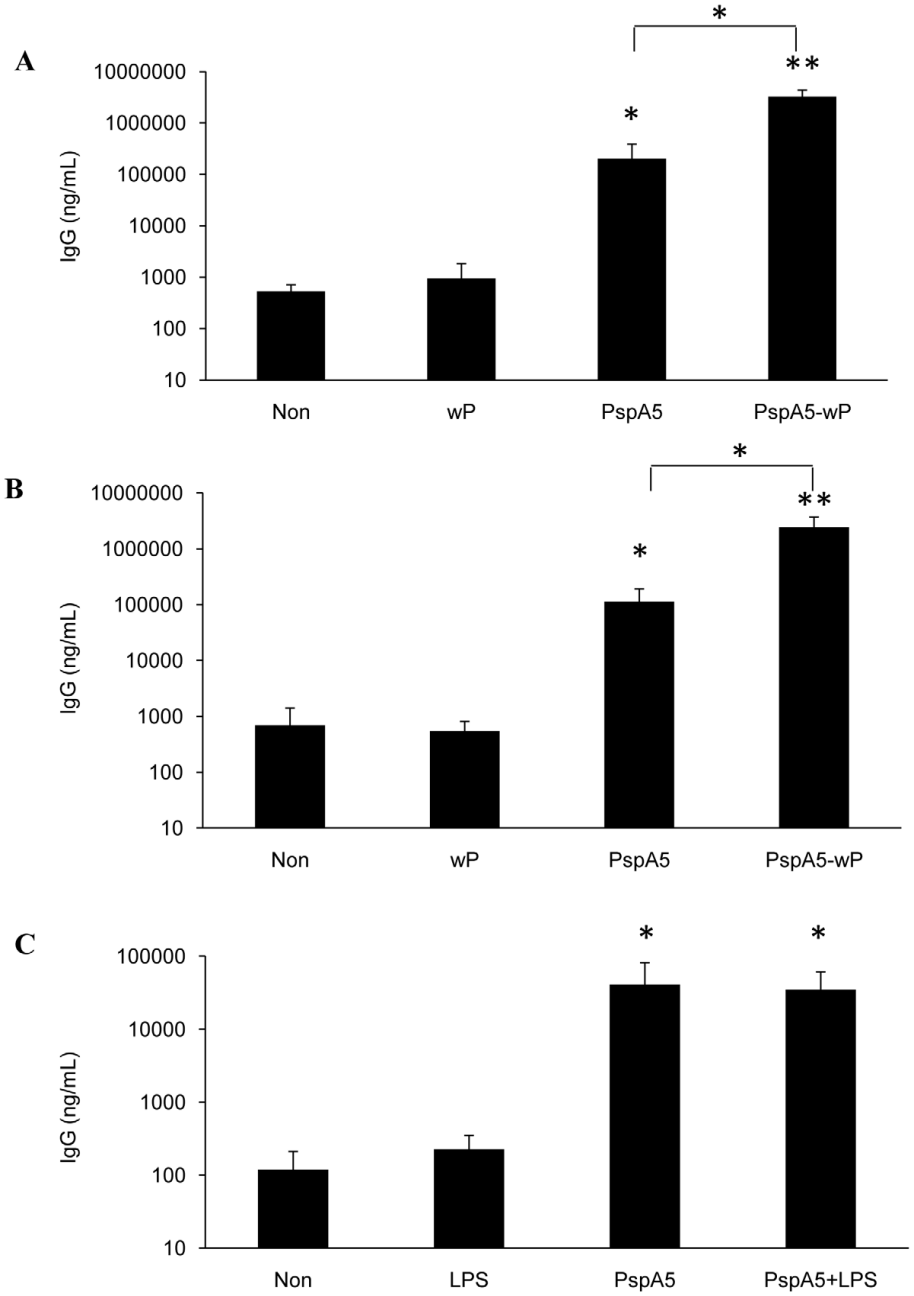
Induction of anti-PspA5 antibodies in C3H/HePas and C3H/HeJ mice. C3H/HePas (A) and C3H/HeJ (B and C) were immunized with the different vaccine formulations through the nasal route. Three weeks after the last immunization anti-PspA5 IgG in the sera were detected through ELISA. Concentration of antibodies (mean of 6 animals) is shown. Results are representative of two experiments. Asterisks represent significant differences from control groups or from group immunized with PspA5, when indicated (* *P*<0.05; ** *P*<0.005, Mann-Whitney *U* test).

Since wP_low_ seems to act as adjuvant for PspA5 in a similar way as wP, we decided to analyze the contribution of *B. pertussis* LPS in the protection elicited by the PspA5-wP formulation. C3H/HeJ mice, which display a point mutation in the *tlr4* gene that abrogates the receptor activity, were immunized through the nasal route as described for BALB/c mice. C3H/HePas mice were used as control mice strain for this experiment. Nasal immunization with PspA5-wP induced significant higher anti-PspA5 IgG in sera from both mice strains, when compared with PspA5 alone (Figure 3A and B). Additionally, significant protection was observed for both mice strains when animals were vaccinated with PspA5-wP (Table 3, *P* = 0.0001, when compared with control groups). To elucidate if *B. pertussis* LPS could act as adjuvant in this model through the activation of other receptors, such as TLR2, we decided to perform an experiment using purified LPS from the *B. pertussis* strain used in our work, in combination with PspA5 in C3H/HeJ. Nevertheless, nasal immunization of mice with PspA5+LPS elicited similar levels of anti-PspA5 IgG as immunization with PspA5 alone (Figure 3C). This formulation did not confer protection against the respiratory pneumococcal challenge in C3H/HeJ mice (Table 4).

**Table 3:** Survival of C3H/HePas and C3H/HeJ mice after intranasal challenge with *S. pneumoniae* ATCC6303.

C3H/HePas	alive/total	% of survival	*P* *
Non-immunized	1/9	11.1	---
wP	2/10	20	0.54
PspA5	4/10	40	0.18
PspA5- wP	10/10	100	0.0001
**C3H/HeJ**			
Non-immunized	0/9	---	---
wP	0/10	---	---
PspA5	4/10	40	0.054
PspA5- wP	9/10	90	0.0001

* Fisher exact test.

**Table 4:** Survival of C3H/HeJ mice after intranasal challenge with *S. pneumoniae* ATCC6303 - combination of PspA and *B. pertussis* LPS.

	alive/total	% of survival	*P* *
Non	0/7	---	---
LPS	0/7	---	---
PspA5	4/7	57	0.03
PspA5 + LPS	2/7	28.5	0.23

* Fisher exact test.

### Passive immunization of mice with sera from animals vaccinated with PspA5-wP protects mice against pneumococcal respiratory challenge with the ATCC6303 strain

**Figure 4:**
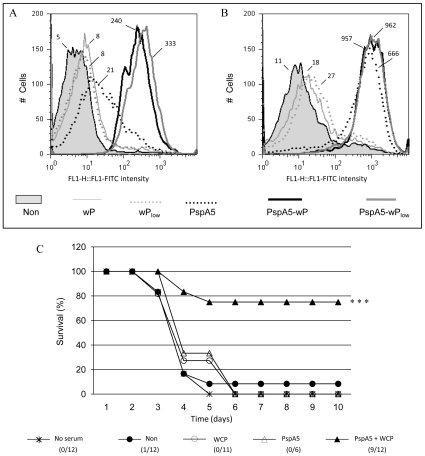
Binding to pneumococcal surface, complement deposition and passive protection elicited by anti-PspA5 antibodies. Sera from BALB/c mice immunized with PspA5 (dotted black lines), PspA5-wP (solid black lines) or PspA5-wP_low_ (solid dark gray lines) were tested for the ability to bind to the pneumococcal surface (A) and to mediate C3 deposition (B). *S. pneumoniae* ATCC 6303(PspA5) was incubated with 2% (A) or 10% of each group serum (B). Sera from non-immunized animals (gray areas) and immunized with wP (solid light gray lines) or wP_low_ (dotted light gray lines) were used as control. The median fluorescence of bacteria is shown for each sample. Data are representative of two independent experiments. Naïve mice were inoculated trough the intraperitoneal route with a 1∶100 dilution of each serum 2 h before pneumococcal challenge. Survival was monitored until 10 days after challenge (C). ***P<0.001 when compared with control groups and *P* = 0.004 when compared with mice vaccinated with PspA5 by Fisher exact test. Data were composed with two independent experiments.

The capacity of each serum to bind to pneumococcal surface was evaluated by Flow Cytometry, using intact ATCC6303 bacteria. The fluorescence intensities were higher when bacteria were incubated with sera from mice immunized with PspA5-wP and PspA5-wP_low_ vaccines, when compared with sera from mice immunized with PspA5 (Figure 4A), correlating with antibodies levels. On the other hand, *in vitro* complement deposition on bacteria surface was similar for sera from mice immunized with PspA5 or the combinations PspA5-wP and PspA5-wP_low_ (Figure 4B).

Since several days were necessary for complete pneumococcal clearance in lungs, but bacteria did not reach the bloodstream of vaccinated mice (Figures 1E and F), we tested whether sera from immunized mice could confer any protection against pneumococcal infection. Naïve mice received pooled sera collected from different groups, as indicated in Figure 4C, in a 1∶100 dilution, by intraperitoneal route, 2 h before challenge. The results showed a significant protection induced by sera from the PspA5-wP group, resulting in 75% of mice survival (Figure 4C).

### Increased anti-PspA antibodies levels induced by the combination with wP improves cross-reactivity against different PspAs

**Figure 5:**
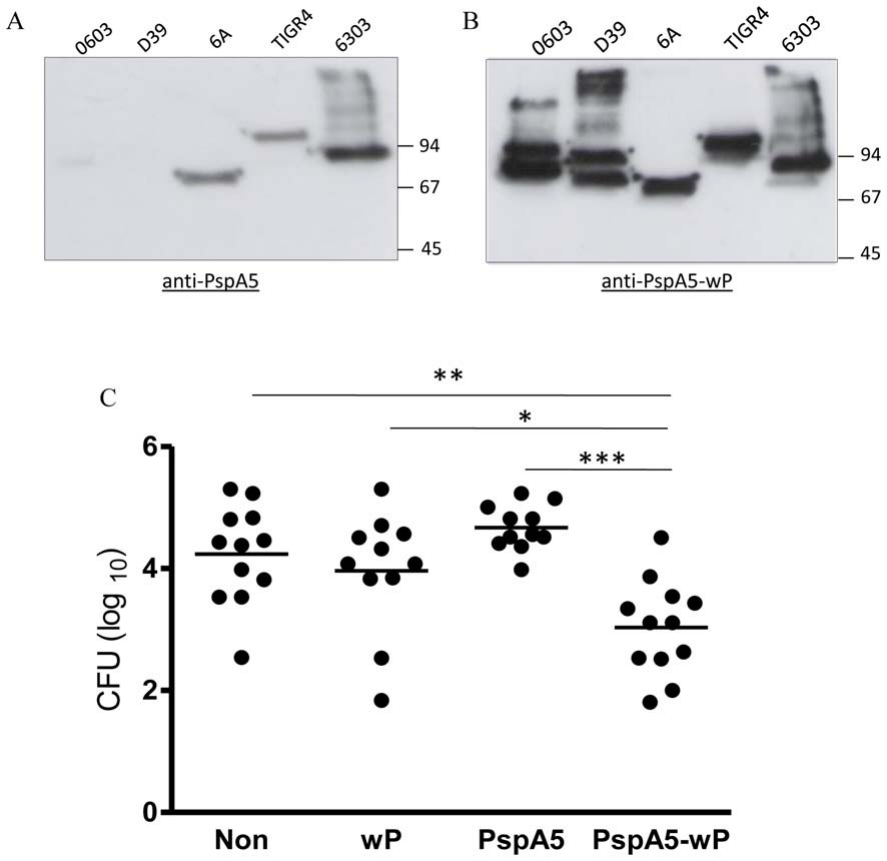
Cross-reactivity and cross protection induced by immunization of mice with PspA5-wP. Western-blot analyses were carried out using total protein lysates from different pneumococcal strains and a 1∶500 dilution of sera from BALB/c mice immunized with PspA5 (A) or PspA5-wP (B). Mice were immunized through the nasal route with the different indicated vaccines. Three weeks after the last immunization mice were challenged with the 0603 pneumococcal strain (PspA1) through the nasal route and CFU were determined in nasal washes, 5 days after challenge. Circles represent individual pneumococcal loads and lines represent the mean for each mice group. Asterisks indicate significant differences when compared with indicated groups (*P = 0.01; **P = 0.003; ***P = 0.001, Mann-Whitney *U* test). Results were composed with two independent experiments.

It has already been demonstrated that sera against the PspA5 fragment used in this work displayed cross reactivity with PspAs from different clades and families [44], [45]. In a panel of strains commonly used in animal models of challenge, we confirmed these results by western-blot of total pneumococcal protein extracts using sera from mice immunized with PspA5 through the nasal route, in the absence of adjuvants (Figure 5A). However, as can be observed in Figure 5B, cross-reactivity was greatly improved in sera from mice immunized with PspA5-wP.

Protection against pneumococcal colonization induced by different PspA-based vaccines has already been described [23], [48]. In order to address the cross reactivity induced by the PspA5-wP vaccine, we have decided to test its efficacy in a well established model of nasopharyngeal colonization in C57Bl/6 mice with the 0603 pneumococcal strain, which expresses PspA from clade 1. Immunization of C57Bl/6 mice resulted in the induction of high levels of anti-PspA antibodies, as observed for the other mice strains (data not shown). Moreover, nasal immunization of mice with PspA5-wP conferred significant protection against nasopharyngeal colonization of mice with the 0603 pneumococcal strain, (*P* = 0.003; 0.01 and 0.001, when comparing PspA5-wP vaccinated mice with non-immunized mice, or mice immunized with wP or PspA5, respectively) (Figure 5C).

**Figure 6:**
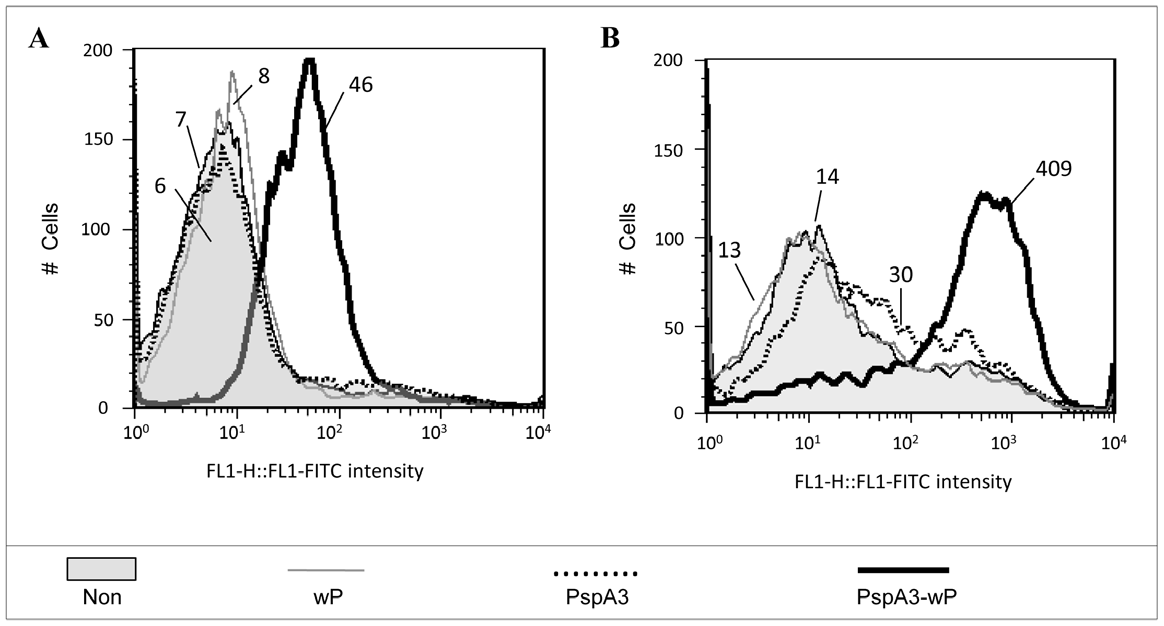
Cross-reactivity induced by immunization of mice with PspA3-wP. Sera from mice immunized with PspA3 (dotted black lines) or PspA3-wP (solid heavy black lines) were tested for the ability to bind to the pneumococcal surface (A) and to mediate C3 deposition (B). *S. pneumoniae* ATCC 6303 (PspA5) was incubated with 2% (A) or 10% of each group serum (B). Sera from non-immunized animals (gray areas) and immunized with wP (solid light gray lines) were used as control. The median fluorescence of bacteria is shown for each sample. Data are representative of two independent experiments.

We then decided to test the effects of wP when using a PspA3 N-terminal fragment, a molecule previously defined to induce sera with low or no cross-reactivity against PspAs from different clades [44], [45]. Mice were immunized through the nasal route with PspA3 or PspA3-wP, as described for PspA5, and sera were collected for analysis. Flow cytometry experiments using the ATCC6303 strain, which expresses PspA from clade 5, showed an improvement in binding capacity for sera from mice immunized with PspA3-wP (Figure 6A). These sera also induced *in vitro* complement deposition on the surface of ATCC6303 pneumococcal strain, whereas sera from mice immunized with PspA3 did not (Figure 6B). Finally, the cross reactivity observed *in vitro* correlated with superior protection of mice after the lethal challenge with the ATCC6303 strain. Hence, while immunization of mice with PspA3 conferred survival to only 30% of mice, the combination PspA3-wP induced 60% of survival (*P* = 0.007, when compared with non-immunized mice) (Table 5).

**Table 5:** Survival of BALB/c mice after intranasal challenge with *S. pneumoniae* ATCC6303 - Combination of PspA3 and wP.

	alive/total	% of survival	*P* *
Non	0/9	---	---
wP	0/10	---	---
PspA3	3/10	30	0.12
PspA3-wP	6/10	60	0.007

* Fisher exact test.

### Combination of PspA5 with the DTP_low_ vaccine protects mice against the respiratory lethal challenge with different pneumococcal strains

**Figure 7:**
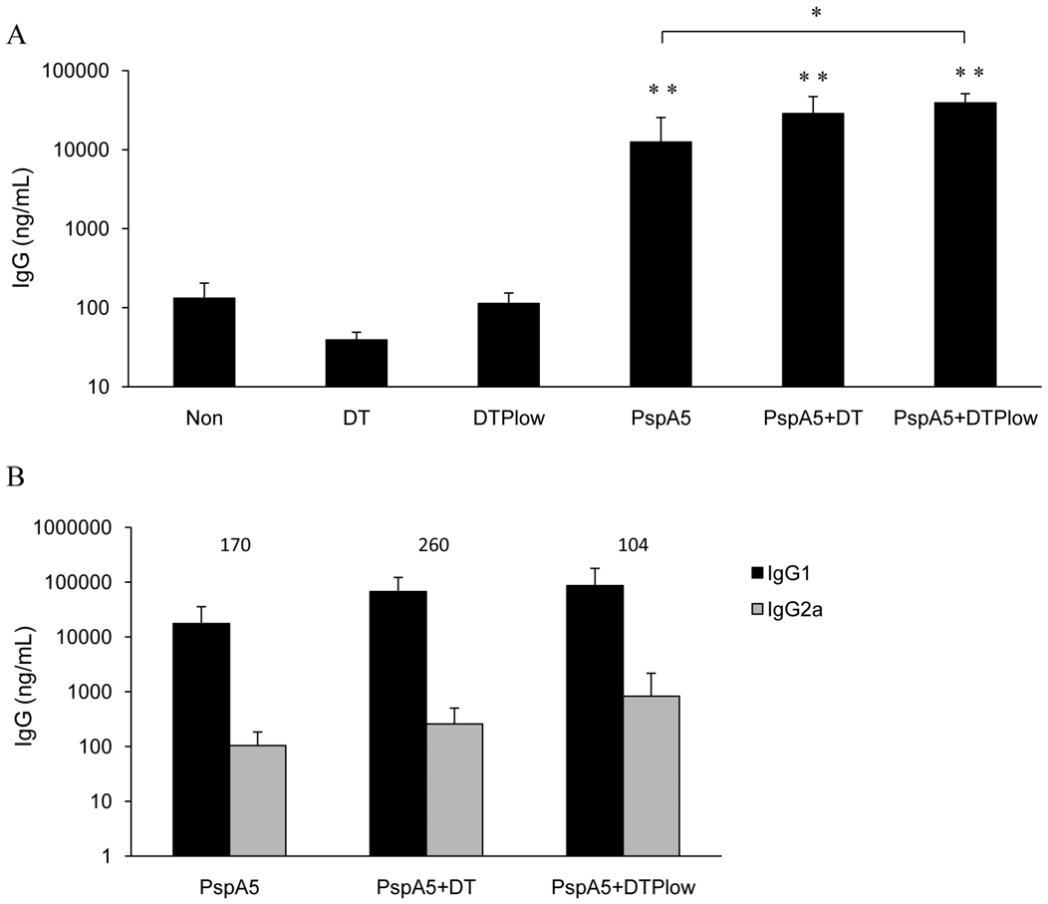
Evaluation of anti-PspA5 antibodies in mice immunized with the different vaccine formulations. Three weeks after the last immunization anti-PspA5 IgG (A), IgG1 and IgG2a (B) in the sera were detected through ELISA. Concentration of antibodies (mean of 6 animals) is shown. IgG1∶IgG2a ratios are indicated above the bars (B). Results are representative of two experiments. Asterisks represent significant differences from indicated groups (**P<0.005, when compared with non-immunized mice or with the respective control groups and *P<0.01, when compared with mice immunized with PspA5, Mann-Whitney *U* test).

Since our findings supported the use of PspA in combination with cellular pertussis vaccines for protection against pneumococcal infections, we decided to test the feasibility of using a combination of PspA5 with the DTP_low_ vaccine in mice. After one single subcutaneous dose composed of 5 µg of PspA5 alone or in combination with DT or DTP_low_ (Figure 7A), higher levels of anti-PspA5 IgG were observed in mice sera, when compared with the respective controls. The formulation PspA5-DTP_low_ also induced significantly higher levels of antibodies when compared with PspA5 alone. Analysis of IgG subtypes showed that the PspA5-DTP_low_ formulation induced slightly higher IgG2a levels than the other vaccines, resulting in lower IgG1∶IgG2a ratio (Figure 7B). Most important, 100% of mice survival, after challenge with the ATCC6303 strain, was observed in the group immunized with one dose of the PspA5-DTP_low_ vaccine (*P* = 0.001, when compared with control groups) (Table 6). Significant protection was also observed for the PspA5-DT formulation, leading to 66% of mice survival (*P* = 0.03, when compared with control groups) (Table 6). Mice immunized with the same formulations were also challenged with the A66.1 strain, which expresses PspA2. In this case, significant protection (66.6% of survival, P = 0.03, when compared with control groups) was only observed in the group immunized with PspA5-DTP_low_ (Table 6).

**Table 6:** Survival of BALB/c mice after intranasal challenge with *S. pneumoniae* ATCC6303 or A66.1 strains, after subcutaneous immunization.

	ATCC6303 (serotype 3, PspA5)			A66.1 (serotype 3, PspA2)		
	alive/total	% of survival	*P* *	alive/total	% of survival	*P* *
Non	0/6	---	---	0/5	---	---
DT	0/6	---	---	0/6	---	---
DTP_low_	0/6	---	---	0/6	---	---
PspA5	2/6	33.3	0.22	0/6	---	---
PspA5 + DT	4/6	66.6	0.03	3/6	50	0.09
PspA5 + DTP_low_	6/6	100	0.001	4/6	66.6	0.03

* Fisher exact test.

## Discussion

In countries where PCV7 vaccine was introduced, a significant reduction in diseases caused by the pneumococcal serotypes included in its formulation was observed [5], [6], [7]. However, after some years of its use, the emergence of new serotypes as prevalent causes of diseases was observed. Although this effect may not exclusively be related to vaccination [49], novel conjugated vaccines composed of more polysaccharides turned out to be necessary for a sustained broad-coverage. Another important consideration is that coverage varies in different parts of the world. A recent study conducted in children attending day-care centers in the city of Goiania, Brazil, estimated that serotype coverage for PCV7 and PCV10 would be around 60%, whereas PCV13 introduction would result in 72% of serotype coverage. Undoubtedly, these numbers are higher in other places of the world [9], [10]. These characteristics, associated with the high costs of the conjugated vaccines are obstacles for their implementation in developing countries.

PspA is a variable molecule present on the pneumococcal surface. However, the distribution of PspA clades in pneumococcal isolates from different regions seems to be similar [50], [51] with an incidence of almost 50% of PspAs belonging to family 1 and 50% to family 2. PspA from family 3 is rarely present in pneumococcal isolates. Usually, formulations are proposed for PspA vaccines based on cross-reactivity among clades from the same family, resulting in the inclusion of at least one component of each family or fusion proteins [19], [52]. In this study, we have chosen to work with PspA5, based on the cross-reactivity and cross-protection results previously described by our group [44], [45].

Whole-cell pertussis vaccine was shown to induce specific T cell responses and mucosal and systemic antibodies against pertussis antigens after nasal immunization of human volunteers [33]. The adjuvant properties of such vaccines also favor the combination with other antigens, which could result in polyvalent formulations that could confer protection against multiple infectious diseases, with reduced number of injections. Immunization of mice with *B. pertussis* cellular vaccines (alone or in DTP formulations) in combination with influenza vaccines was shown to induce high levels of antibodies against influenza and antigen-specific IL-2 and IFN-γ secretion [37], [53], [54]. A combination of DTPw and a Hepatitis B vaccine (HBV) resulted in higher humoral response to hepatitis in children in Slovakia, when compared with children that received the HBV alone [55]. Due to the adjuvant properties of DTPw, equivalent responses to *Haemophilus influenzae* type b conjugate vaccine (Hib) were observed in children that received the vaccine alone or 1/10 of the dose directly diluted in DTPw [56]. Simultaneous administration of PCV7 with a DTPw/IPV-inactivated polio virus-/Hib vaccine was also shown to produce satisfactory immune responses to pneumococcal polysaccharides in French infants [57]. More detailed studies using an 11-valent pneumococcal vaccine have demonstrated a reduced response to pneumococcal polysaccharides when the vaccine was simultaneously administered with a DTPa/IPV/Hib vaccine. This effect was not observed when the pertussis cellular component was used [58]. In the present work, we have combined the PspA5 antigen with a whole cell pertussis vaccine (wP), which is the pertussis component of the current Brazilian DTPw vaccine, for tests as a nasal formulation in mice. A robust protection against a lethal respiratory challenge with the pneumococcal ATCC6303 strain was observed in mice immunized with PspA5-wP.

Different cellular components are responsible for the adjuvant effects of wP and are also believed to contribute to the adverse effects observed [59], [60], [61]. Differences in LPS amounts among vaccine formulations as well as the inactivation process during vaccine manufacturing were some of the hypothesis raised to explain differences in reactogenicity displayed by wP vaccines produced by different countries. Still, controversies on this issue persist to date and DTPw is administered to children in several developing countries, inducing high levels of protection against pertussis [38]. In an attempt to improve the acceptability of wP vaccine, Instituto Butantan (São Paulo, Brazil) has developed a methodology to remove the excess of LPS present in wP, producing the wP_low_. In the present work, we have also tested the efficacy of wP_low_ as a mucosal adjuvant for PspA5. Similar protection levels were observed for the PspA5-wP_low_ vaccine, when compared with PspA5-wP. Overall, the immune responses induced by both formulations seemed quite similar, with controlled inflammatory responses elicited in lungs (manuscript in preparation). This led us to test the role of *B. pertussis* LPS as the determinant adjuvant component in our model. Combination of wP with PspA5 induced significantly higher anti-PspA5 antibody concentrations in C3H/HePas and C3H/HeJ mice than PspA5 alone, indicating that the adjuvant activity was preserved in both mouse strains. Moreover, in both cases, nasal immunization with PspA5-wP induced protection against the lethal challenge with the ATCC6303 strain. TLR4 responses were already shown to be important for Th1 and Th17 responses induced by wP [32], [62]. Conversely, purified *B. pertussis* LPS (properly named by the authors as Lypooligosaccharide – LOS) were shown to induce *in vitro* Th2-biased immune responses via TLR4 in human cells [63]. Monophosphoryl lipid A derived from the same *B. pertussis* strain used in this work has already been shown to be a good adjuvant for influenza vaccine [64]. It is possible that the residual LPS present in wP_low_ contributes to the adjuvant properties and overall protection observed in wild type mice; however, immune responses directed to PspA5 in the absence of TLR4 are enough to confer protection against the pneumococcal challenge. Other components such as the Pertussis Toxin or the adenylate cyclase toxin may be also acting as adjuvants to PspA5. The possibility that the LPS derived from our *B. pertussis* strain could be activating receptors other than TLR4 seemed not to be the case, since nasal immunization of C3H/HeJ mice with PspA5 + LPS neither increased the antibody levels induced by PspA5 alone nor protected mice against pneumococcal challenge. In fact, it has already been shown that LPS derived from other *B. pertussis* strains stimulates TLR4 responses but are not able to induce TLR2 responses [63], [65].

In BALB/c mice immunized through the nasal route with both PspA5-wP and PspA5-wP_low_, pneumococci were restricted to the lungs. Sera from immunized mice presented high levels of anti-PspA5 IgG, more balanced IgG1∶IgG2a ratios and induced *in vitro* complement deposition on pneumococcal surface. These conditions were previously associated with increased protection elicited by DNA vaccines composed of PspA [15] or intranasal vaccines composed of PspA and IL-12 [24]. We thus decided to analyze if the sera induced by PspA5-wP could elicit protection against the pneumococcal lethal challenge. Passive immunization experiments conducted in BALB/c mice have shown that the antibodies produced by nasal immunization with PspA5-wP conferred protection to naïve mice against the pneumococcal challenge. This result suggests that although cellular responses can improve the quality of the immune response against the respiratory pneumococcal challenge in mice, leading to better protection, a condition where high levels of anti-PspA5 antibodies are present can be sufficient to induce significant protection. The high levels of antibodies induced by the combination of wP with PspA5 also reflected in increased cross-reactivity of the sera against PspAs from different strains and clades. As a result, significant protection against pneumococcal nasal colonization by a strain that expresses PspA from clade 1 was also observed in C57Bl/6 mice immunized with PspA5-wP through the nasal route. Moreover, combination of wP with a PspA from clade 3, which was previously shown to induce antibodies with poor or no cross-reactivity against PspAs from other clades [44], [45], greatly improved serum reactivity against the ATCC6303 strain, increased complement deposition onto bacterial surface and induced significant protection.

Several groups have proposed the nasal immunization of wP as an alternative to decrease the possible side effects elicited by the vaccines in infants or as strategies for boosters in young adults. The present work shows that wP can also be a potent adjuvant to PspA, when administered through the nasal route, conferring protection against pneumococcal infection in mice. However, approved DTPw vaccines are administered to children through the intramuscular route. For this reason, we decided to analyze if DTP_low_, a new generation DTPw vaccine, could also improve immune responses and confer protection against pneumococcal infection in mice, when combined with PspA5. We have observed that a single immunization of mice with PspA5-DTP_low_ through the subcutaneous route induced 100% survival against the challenge with the ATCC6303 strain (which expresses PspA5) and 66.6% survival against the challenge with the A66.1 strain (which expresses PspA2). Improved protection against ATCC6303 strain was also observed when PspA5 was combined to DT, although in a lesser extent than the observed for the PspA5-DTP_low_ combination. In a previous publication we have shown that immunization of mice with the same PspA5 fragment, in combination with Al(OH)_3_ as adjuvant (which induces a Th2 response), resulted in 67% of protection against the respiratory challenge with pneumococcal ATCC6303 strain [45]. This is very similar to the protection observed when we combined PspA5 with DT (66.6% protection, Table 6) which also has Al(OH)_3_ in its formulation. It has already been shown that optimal responses against pneumococci are achieved with PspA-based vaccines that induce balanced IgG1∶IgG2a levels [15], [24] and we propose here that wP is an adequate adjuvant to induce such responses. Prime-boost protocols in which PspA5-DTP_low_ and PspA5-DT are combined can be tested and this also opens the possibility of inclusion of PspA5 in the DT vaccine given to adults.

Based on the results presented here, we propose that the inclusion of a PspA molecule, defined by its ability to induce cross-reactive antibodies against different PspAs, in the current DTPw vaccine, constitutes an efficient strategy for a new vaccine against pneumococcal infections, with broad-coverage and low-production cost, for vaccination of children in developing countries. Experiments are now being conducted to determine if the inclusion of PspA5 would produce any effect on the immune responses against the DTP antigens. Finally, in an attempt to reproduce the immunization calendar of infants from developing countries we will now study the inclusion of BCG in this model.

## Materials and Methods

### Ethics statement

This work was performed in mice. During all experiments, animals were supplied with food and water *ad libitum* and experimental protocols were previously approved by the Ethical Committee for Animal Research of Butantan Institute, under the license number 457/08.

### Bacterial strains and growth conditions

*S. pneumoniae* strains were grown in Todd-Hewitt broth (Difco) supplemented with 0.5% yeast extract (THY), at 37°C, without shaking. Bacteria were always plated in blood agar and grown overnight at 37°C before inoculation in THY. Stocks were maintained at −80°C in THY containing 20% glycerol. The ATCC6303 strain (serotype 3, PspA clade 5) was a kind gift from Dr. Maria Cristina Brandileone (Instituto Adolpho Lutz, São Paulo, Brazil) and the A66.1 strain was kindly provided by Dr. David E. Briles (University of Alabama at Birmingham, USA). Both strains were used for the lethal respiratory challenge. For colonization experiments, pneumococcal strain 0603 (serotype 6B, PspA clade 1) was a gift from Dr. Richard Malley (Childreńs Hospital, Harvard Medical School, MA, USA). For Western blot analysis, the pneumococcal strains D39 (serotype 2, PspA clade 2) kindly provided by Dr. David E. Briles, P2139 (serotype 6A, PspA clade 2) [66] and TIGR4 (serotype 4, PspA clade 3) both kindly provided by Dr. Jeffrey N. Weiser (University of Philadelphia School of Medicine, USA) were used, in addition to ATCC6303 and 0603 strains.

### Recombinant proteins and vaccine formulations

The N-terminal fragments of PspA from clade 5 and 3 (from strains 122/02 and 259/98, respectively – Instituto Adolpho Lutz, São Paulo, Brazil), were expressed in *E. coli* BL21 SI (Invitrogen) and purified by chromatography as previously described [44]. The whole cell pertussis vaccine (wP) used in this work is composed of the whole bacteria inactivated with 0.2% formalin and is currently produced by Instituto Butantan (São Paulo, Brazil). This vaccine is used for the formulation of the DTPw vaccine (composed of Diphtheria and Tetanus toxoids and inactivated whole cell pertussis, in the presence of Al(OH)_3_). The DTPw produced by Instituto Butantan is currently distributed by the Brazilian Ministry of Health to children all over the country, for vaccination at 2, 4 and 6 months of age. The wP_low_ is a new generation cellular pertussis vaccine produced by Instituto Butantan by the removal of great part of the *B. pertussis* LPS through extraction with organic solvent and is the pertussis component of the DTP_low_ vaccine that has just passed through a phase 1 clinical trial [46].

### Immunization of mice and antibody responses

BALB/c mice were produced by the animal facility from Universidade Federal de São Paulo (CEDEME). C3H/HePas and C3H/HeJ mice were produced by the animal facility from Faculdade de Medicina Veterinária da Universidade de São Paulo. C57Bl/6 mice were provided by the animal facility from Instituto Butantan. For the lethal pneumococcal challenge model, immunization was performed in groups of 4 to 6 female BALB/c mice and 6 to 10 C3H/HePas and C3H/HeJ mice (males and females). Each vaccine dose contained 5 ug of PspA5 or PspA3 alone or in the presence of 1/8 of the Human wP, wP_low_, DT (Diphtheria and Tetanus and toxoids in a formulation that contains Al(OH_3_)) or DTP_low_ (described above) doses. In these conditions, each dose of wP, wP_low_ or DTP_low_ contained 0.5 mg of total pertussis mass. In some experiments, purified *B. pertussis* LPS (10 µg/dose - produced by Instituto Butantan) was also combined with PspA5. Before formulation of all vaccines tested, residual LPS from *E. coli*, present in PspA5 and PspA3 preparations was removed by triton X-114 extraction as previously described [67]. Nasal immunization was conducted in mice previously anesthetized through the intraperitoneal (i.p.) route with 200 µL of a 0.2% xilazine and 0.5% ketamine mixture. Vaccines were administered in a volume of 10 µl on days 0, 3, 14, 17, 28 and 31 (total of 6 doses). For subcutaneous immunization, mice received 1 dose of each vaccine in a volume 100 µl. For the pneumococcal colonization model, groups of 6 female C57Bl/6 mice were immunized through the nasal route as described above. In all experiments, control groups that received only the adjuvants or that were not immunized were included. Mice were bled through the retrorbital plexus fifteen days after the last immunization. Serum and bronchoalveolar lavage fluid (BALF) (collected as described below) were evaluated for antibody levels were by ELISA in plates coated with PspA5. The assay was performed using goat anti-mouse IgG, IgA, IgG1 or IgG2a and rabbit anti-goat conjugated with HRP (Southern Biotech). Standard curves were generated using mouse IgG, IgA, IgG1 and IgG2a (Southern Biotech).

### Intranasal pneumococcal challenges and passive immunization experiments

For intranasal respiratory lethal challenge, immunized mice were anesthetized through the intraperitoneal route with 200 µL of a 0.2% xilazine and 1.0% ketamine mixture. ATCC6303 or A66.1 pneumococcal strains were grown in THY until mid-log phase (OD_600nm_ = 0.4) and aliquots were maintained at −80°C until use. Animals were challenged 21 days after the last immunization with 3×10^5^ CFU/animal (ATCC6303) or 1×10^6^/animal (A66.1) in 50 µL of saline, inoculated into one nostril of mice, with the help of a micropipette. Animals were observed for 10 days for survival records. For passive immunization experiments, sera collected from immunized mice were pooled and inactivated at 56°C for 30 min. Groups of 6 naïve BALB/c mice were inoculated intraperitoneally with 500 µL of a 1∶100 dilution of each serum, 2 h prior challenge with the ATCC6303 strain. Animals were observed for 10 days for survival records.

For nasopharyngeal colonization experiments, the 0603 strain was also grown in THY until OD_600_nm = 0.4 and aliquots were maintained at −80°C until use. Twenty one days after the last immunization, C57Bl/6 mice were anesthetized through the intraperitoneal route with 200 µL of a 0.2% xilazine and 0.5% ketamine mixture and received 5×10^6^ CFU in 10 µL of saline, into both nostrils. Mice were sacrificed 5 days later and pneumococcal loads were determined in nasal washes performed as previously described [68], by plating serial dilutions of the samples in blood agar and incubating the plates at 37°C for 18 h.

### Bronchoalveolar lavage fluid (BALF) collection and recovery of bacteria from blood and lungs

Twenty one days after the last immunization dose, mice were sacrificed through injection of a lethal dose of urethane (15 mg per 10 g of body weight) to collect BALF samples. A catheter was inserted into the trachea of the mice and lungs were rinsed with 0.5 mL of sterile PBS followed by an additional rinse with 1 mL of PBS. The fluids from both rinses were pooled aliquoted and maintained at −80°C for antibody analysis.

For pneumococcal recovery, immunized mice were sacrificed as described above at different time points after challenge. Lungs from each mouse were collected and disrupted in 1 mL of half saline with the use of a cell strainer. Serial dilutions of lung homogenates or blood (collected through the retroorbital plexus) were plated on blood agar. Plates were incubated overnight at 37°C and CFU determined. Detection of 0 CFU was considered 1 CFU. The limit of detection was 100 CFU/mL in blood samples and 5 CFU/animal in lung samples.

### Antibody binding and complement deposition assays

*S. pneumoniae* ATCC6303 frozen stocks were plated on blood agar overnight then grown in THY to OD_600nm_0.4–0.5 (∼10^8^ CFU/ml) and harvested by centrifugation. Bacteria were washed, suspended in PBS and incubated with 2% of pooled sera during 30 min at 37°C. Samples were washed once with PBS before incubation with fluorescein isothiocyanate (FITC)-conjugated anti-mouse IgG (Sigma) for 30 min on ice. For complement deposition assays, sera were previously heated at 56°C for 30 min and incubated with bacteria at a concentration of 10%, at 37°C for 30 min. Samples were washed once with PBS and incubated with 10% of normal mouse serum as source of complement in Gelatin Veronal buffer (Sigma), at 37°C for 30 min. After washing, samples were incubated with FITC-conjugated anti-mouse C3 IgG (MP Biomedicals) in PBS, for 30 min on ice. Samples were fixed with 2% formaldehyde after two washing steps and stored at 4°C. Flow cytometry analysis was conducted using FACSCalibur (Becton Dickinson), and 10,000 gated events were recorded. The median of fluorescent bacteria were used to compare the groups.

### Western blot analysis

Western-blots were performed with soluble protein lysates from the pneumococcal strains D39, P2139, TIGR4, ATCC6303 and 0603. All strains were grown in THY until OD_600_ = 0.5 and bacteria were collected by centrifugation. Pellets were washed once in PBS and then lysed by the incubation with 1∶10 of the initial volume of a 0.1% sodium deoxycholate/0.15 M sodium citrate solution, for 10 min at 37^°^C, as previously described [69]. Samples were separated by SDS-PAGE and transferred to nitrocellulose membranes. Anti-PspA5 or anti-PspA5-wP antiserum was added in a dilution of 1∶500, followed by incubation with HRP-conjugated goat anti-mouse IgG (1∶1000, Sigma). Detection was performed with a chemiluminescent ECL kit (GE HealthCare).

### Statistical analysis

Differences in survival rates were analyzed by Fisher exact test. Differences in antibody concentrations and pneumococcal loads in nasal colonization experiments were analyzed by the Mann–Whitney *U* test. In all cases, *P*≤0.05 was considered significantly different.
